# Raising double-muscled breed cattle and their crossbreds in the tropics: insight from growth models

**DOI:** 10.14202/vetworld.2024.1504-1513

**Published:** 2024-07-13

**Authors:** Ummi Noorhakimah Abdullah, Goh Yong Meng

**Affiliations:** 1 Department of Veterinary Pre-Clinical Science, Faculty of Veterinary Medicine, Universiti Putra Malaysia, 43400 Serdang, Selangor, Malaysia; 2 Department of Veterinary Services, Ministry of Agriculture and Food Security, 62624 Putrajaya, Malaysia; 3 Institute of Tropical Agriculture and Food Security, Universiti Putra Malaysia, 43400 Serdang, Selangor, Malaysia

**Keywords:** beef cattle, Belgian Blue crossbreds, Brahman, double-muscled, growth performance, Kedah-Kelantan, non-linear regression growth functions

## Abstract

**Background and Aim:**

In tropical conditions, modeling the predictive parameters of live weight, including those at birth, pre-weaning, post-weaning, finishing, and maturing, and the average daily gain, is challenging. The heat load significantly influences the growth rate and final mature weights in the tropics. The study compared the growth rates of Kedah-Kelantan (KK), Brahman (BRAH), and Belgian Blue (BB) crossbred calves.

**Materials and Methods:**

The study conducted growth analysis using the non-linear regression growth models as it approximates the sex, breed, and growth physiology changes in beef cattle. It is supported by the utility of the most common growth functions (Brody, Logistic, von Bertalanffy, and Richard’s model) in normal-muscled tropical breeds and double-muscled crossbred beef cattle in the tropics.

**Results:**

The BB crossbreds outperformed the KK and BRAH breeds by 50%–100% in live weight gains under tropical conditions. The crossbreds display the double-muscled effect and highlight the advantages of heterosis, making them suitable for upgrading local herds. The study’s findings on the growth characteristics of BB crossbred cattle were best described by the von Bertalanffy growth model, which had a high coefficient of determination (R^2^ > 0.8) and yielded estimated mature weights of 527.5 kg for males and 518.5 kg for females.

**Conclusion:**

According to results, raising BB crossbreds in the tropics as a solution to ensure a sustainable beef supply could yield significant growth and economic benefits.

## Introduction

Growth traits are directly related to animal productive life [1] and comprise observable genetic traits and multifactorial environmental elements [2]. Understanding the genes and linked chromosome areas linked to desirable growth traits can significantly aid in evaluating the breeding value of offspring [3]. When determining the breeding value of progeny, knowledge of the genes and chromosome areas linked to the desired overall growth attributes may be highly helpful [3]. The myostatin gene is primarily expressed in developing and mature muscle cells, limiting their growth [4]. A reported myostatin gene mutation impairs muscle growth regulator function, leading to double-muscling [5]. Since *Bos indicus* reports on myostatin gene mutation lack the diversity found in *Bos taurus*, it is crucial to explore the feasibility of using double-muscling in tropical beef cattle breeding. Double-muscled breed produce more meat significantly than other breeds [6]. The prominent double-muscled Belgian Blue (BB) (*B. taurus*) breed [7] boasts a high growth quality [8]. BB cattle often exceed a 60% dressing percentage on average [9]. It was reported that BB cattle frequently average more than 60% dressing percentage [9]. The lean meat content of double-muscled cattle ranges between 20% and 130% higher, while their fat content is 30%–50% lower compared to normal-muscled cattle [10]. Consumers preferring minimal beef fat might opt for the BB breed. Upgrading beef cattle using BB genetic materials in the tropics will significantly boost beef productivity and supply. In the 2000s, BB cattle contributed significantly to elevating Belgium’s beef supply self-sufficiency level above 157%. It would be optimal to improve tropical BB crosses to incorporate superior traits and successfully raise them within resource constraints [11]. There are thousands of different varieties of beef cattle, most of which belong to *B. taurus* or *B. indicus* [12]. Crossbreeding can be a very effective tool, especially when the parents are genetically distant (e.g., *B. taurus* and *B. indicus*), thus producing a crossbred offspring generation that overgrows and possesses better adaptation traits [13], particularly in the tropics [14]. A study conducted in Colombia found that crossbreeding Brahman (BRAH) cattle with BB led to improved growth rates, feed conversion efficiency, and carcass traits without causing adverse effects on calving ease or dystocia rates [15].

The success of introducing growth traits in beef cattle, such as double muscling, can be measured solely through growth analysis using field data. Among smallholder farmers, beef cattle profitability is mainly determined by the final market weight rather than overall growth performance. Evaluation of growth performance in beef cattle farming is crucial because it indicates the potential production level of beef output [16] and the optimal time to market, which invariably differs from breed to breed. Growth performance measurement is challenging because of the need for comprehensive equipment, expertise, personnel, and time investment resources, as well as understanding factors contributing to beef cattle growth [17]. From a physiological point of view, the growth of beef cattle can be divided into three main stages: the calf stage, the growing stage, and the finishing stage [18]. According to Cronjé [19], the calf and growing stages involve simultaneous active development of nerves, bones, muscles, and skin under the influence of both genetics and hormones. Eventually, the quantity of body cells responsible for lean meat and bone production will no longer expand. Fat tissues alone will continue accumulating energy from the diet. The profitability of selling the cattle depends on their rearing costs, which fluctuate based on their liveweight and muscle-to-bone ratio. Non-linear equations provide the most accurate description of growth and body mass accretion in living things, something that linear equations are unable to do [20]. It is also a common technique to examine how an animal grows over time [21]. The method can be used to estimate daily feed requirements or assess how breed affects an animal’s ability to gain weight [22].

A growth model for an animal, accounting for its physiology, features an inflection point signifying the onset of decelerated growth as it approaches the growth plateau [23]. This would enable the model to reflect the animal weight gain in the real-world. In tropical breeds, the growth rate decline occurs earlier due to the combined effects of unfavorable climatic conditions and poor nutrition from tropical pastures. Non-linear regression growth models enable live weight-age (*Yt*) estimation [24]. The primary goal of non-linear regression growth models for cattle is to determine their biological maturity parameters. The growth rate plays a vital role during fattening, determining both feeding quantity and fattening length [25]. At maturity, a higher feed ratio no longer yields economic benefits. Failing to understand the growth dynamics will result in delayed economic recovery and less profitable farming. To identify the most cost-effective beef cattle breeds given their varying growth and maturation rates, farmers need to make informed choices. Knowledge of growth characteristics at maturity would also allow farmers to identify individual cattle with the most significant potential for further breeding selection programs [26].

**Figure-1:**
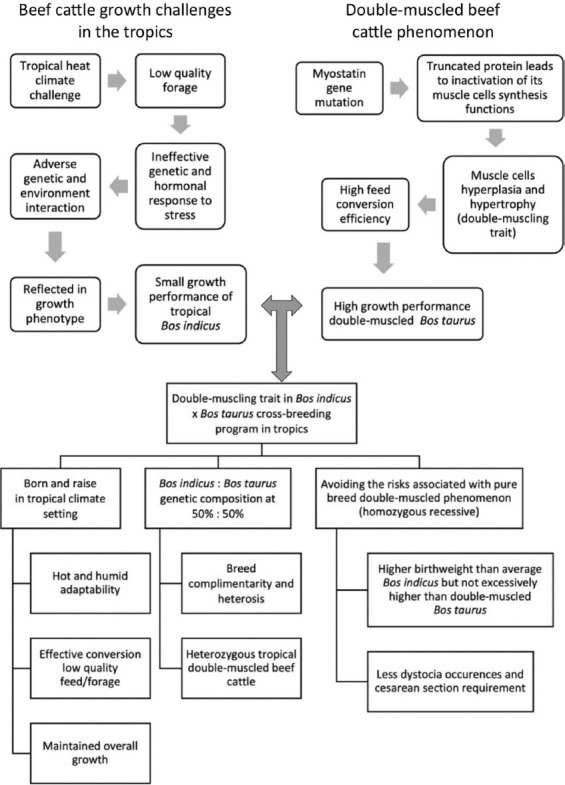
The framework of the research on the utilization of double-muscling traits to improve the overall growth of the *Bos indicus* in the tropics through *Bos indicus* × *Bos taurus* crossbreeding.

Despite being known for their drawbacks, double-muscled crossbred offspring from this study showed exceptional performance. The double-muscled crossbred offspring from crossbreeding hold a competitive edge in the beef cattle market due to their positive net balance of advantages over drawbacks from the double-muscling trait. According to Figure-1, challenges for *B. indicus* in tropical regions include poor forages, scarce feed resources, and genetic factors. Poor nutrients and heat stress hinder growth. Double-muscled *B. taurus*, like BB, can be genetically influenced for accelerated growth. To produce crossbred offspring with higher growth, a double-muscling trait is introduced. The primary objectives are to acclimate to the local climate, maximally convert low-quality feed, maintain a larger live weight, achieve the highest heterosis, and prevent excessive birth weight. The crossbred offspring generation of *B. indicus* and *B. taurus* is anticipated to exhibit greater growth than the average pure breed *B. indicus*, but not exceeding the pure breed double-muscled *B. taurus* growth rate. In Malaysia, the growth of normal-muscled *B. indicus* pure breeds (Kedah-Kelantan [KK], BRAH) and the double-muscled BB crossbreed (*B. taurus* × *B. indicus*) were compared using the best non-linear regression models. The KK and BRAH breeds are the common indigenous, unimproved cattle breeds in Malaysian beef farming. The study’s conclusions, applicable to other tropical regions, can be implemented using different native breeds. Modeling growth stage characteristics in different beef cattle breeds is achieved using non-linear regression growth functions. The economic viability of farming BB crossbred cattle in the tropics can be determined by extrapolating the growth estimation.

This study investigated the potential profitability and feasibility of using the doubling-muscling trait in beef production for tropical regions. The study’s findings enable informed decisions regarding BB genetic materials usage by smallholder farmers, policymakers, and researchers.

## Materials and Methods

### Ethical approval

This study was reviewed and approved by Research Committee, Department of Veterinary Services, Malaysia [Ref: JPV.BPI.600-1/7/1].

### Study period and location

**Figure-2:**
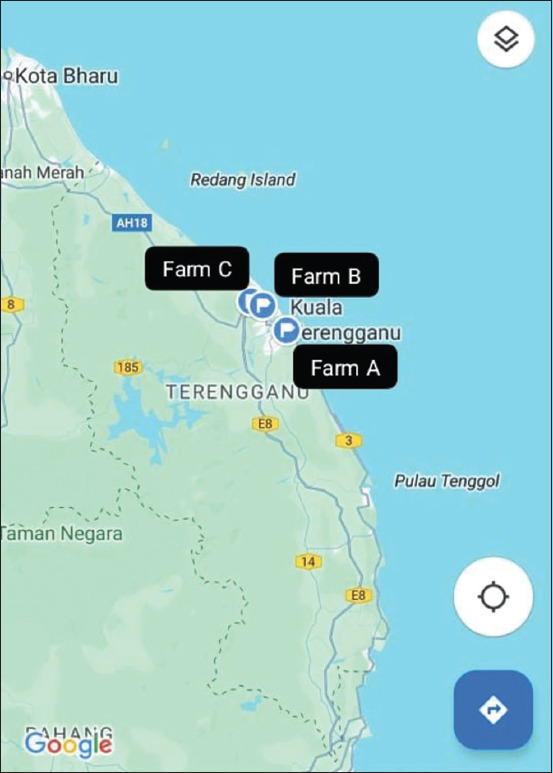
The distributions of Farm A, Farm B, and Farm C located in Terengganu state (5.0936°N, 102.9896°E), East Coast Region of Peninsular Malaysia (source: https://bit.ly/3S2oWI6).

The study period was 18 months (540 days), starting from October 2019 to March 2021 and conducted at Terengganu state, East Coast Region of Peninsular Malaysia. The distribution of the selected farms is displayed in Figure-2.

### Farm background

The current study was implemented considering Malaysia as a representative site of BB farming in the tropics. This is mainly because there is a surge in interest in farming heavier, double-muscled animals for beef production in Malaysia. Malaysian smallholder beef cattle farming was chosen to represent the rearing situation in a hot tropical climate. The offspring were selected randomly from three farms (Farm A, Farm B, and Farm C) located in Terengganu state, East Coast Region of Peninsular Malaysia (Figure-2), with the average annual rainfall, temperature, and humidity of the region being 2750 mm, 28°C, and 83.5%, respectively [27]. Each of the farms was located within a 5 km radius. Beef cattle farming in Terengganu is a popular activity among the local community, with most farmers running the farm as a traditional family business. The selected breed varies, but a double-muscled breed like BB has gained a place among the local smallholder farmers to boost the liveweight performance of their local beef cattle herd through crossbreeding. Terengganu is also one of the biggest palm oil plantation producers in Malaysia Field [28]; hence, palm oil by-products are essential feed resources for the local beef cattle farms. Farm A is a governmental nucleus herd comprising only KK purebreds. At the same time, Farm B is a governmental nucleus herd comprising only BRAH purebreds. The combined 31 BB crossbred smallholder farms represented Farm C. The BB crossbred herd was born from the artificial insemination (AI) program of the KK and BRAH cows with good body condition score (4–5, Likert scale) and BB pure breed frozen semen. A smallholder farm was defined as raising no more than 20 beef cattle at once. The farm operation was commonly carried out in the house’s backyard; hence, there was minimal space to grow the herd. A few small barns under the trees were set up to be used as a shading area during hot days, and basic self-made cattle crate was attached to the trees to secure the cattle for AI or health and treatment program. From birth until weaning (180 days of age), the calves consumed only milk from the dam. The post-weaning feeding routine of farms A, B, and C was the same and was comprised of *Brachiaria humidicola* grass (grazing or cut-and-carry) and palm kernel cake (PKC) concentrates, based on the dry matter intake (DMI) percentage, which is equivalent to 3% of the current liveweight. Due to financial constraints, no other supplementation was provided. The cattle were managed under a semi-intensive system on all farms, with foot and mouth disease vaccination program imposed twice a year and deworming program was based on selected protocol.

### Data collection

A total of 40 male and 40 female offspring were recruited from Farm A (KK) and Farm B (BRAH). In contrast, for the BB crossbred offspring at Farm C, 138 animals, comprising 72 males and 66 female calves, were included in the current study. Only offspring born in September 2017 were selected to ensure a corresponding age-wise comparison. In total, 298 animals from these three farms were included in the study. Liveweight measurements were conducted starting from birth until the age of 540 days (18 months). Two personnel from each farm were appointed to conduct the measurements and keep records. From birth until weaning, liveweight measurements were performed using a calf sling and hanging scale (Rural365, U.S., weighing capacity: 2–300 kg) for improved precision. After weaning, all live weight measurements were conducted using a calibrated metal weighbridge (Algen Scale Corporation, New York, U.S., weighing a maximum capacity of 1000 kg) installed at the farm. It consisted of a pair of load bars set up under a platform placed on a firm and even surface to prevent instability. The load bars were then connected to the scale standing display beneath the platform to avoid being walked on, chewed on, or squashed by the cattle. The cattle then moved onto the platform with proper restraining, and the liveweight reading was recorded from the display board once it stopped blinking. The same personnel weighed calves every 30 days before the daily feeding time at 8 a.m. to minimize error and variation.

### Statistical analysis

The liveweight data were fitted into a non-linear regression model using the Statistical Package for the Social Sciences® software (IBM Corp., NY, USA). In non-linear regression, biological growth parameters are parts of the model that the procedure estimates. It must be defined and named with a valid variable name. It should appear in the model with its starting values. The starting values of the parameters are the figures that are as close as possible to the expected final valuation. It is vital to determine good starting values to avoid failure of iterative convergence [29]. The model expression factors and parameters considered in this study are depicted from the model equations in Table-1. Thus, the parameters *A*, *B*, *k*, and *e* represent parts of the model estimates that must be defined and named first. In the beef cattle growth study [23], the inflection point is where the liveweight of the cattle starts to enter the mature stage or is interpreted as the mature liveweight (*A*) at the proportion of (*B*) that can be reached after birth [21]. The maturing rate of (*k*), where *e* is the exponential value, promotes the curve’s self-acceleration [30] and the maturing index that determines the rate at which liveweight approaches maturity [23]. It is a constant variable directly related to the postnatal maturing rate [22]. The starting values entered for both sexes for A were 200 (Farm A), 500 (Farm B), *B* was 0.1 (both farms), and *k* was 0.01 (both farms). For Farm C (both sexes), the starting values for A were 500, B were 0.1, and *k* were 0.01. The growth analysis for all farms was then continued by retrieving the coefficients of *A*, *B*, and *k* (parameter estimates), the correlation of determination (R^2^), and the residual mean square error (MSE) value from the SPSS output sheet. The retrieved value was the closest estimate derived or generated using the SPSS non-linear regression model procedure. The analysis was conducted separately for Brody’s, Logistic’s, von Bertalanffy’s, and Richard’s models. The best-fit model was chosen if it had the highest R^2^ and lowest MSE residual value.

**Table-1:** The non-linear regression growth model with the equation functions.

Growth model	Equation
Brody	*Yt* = *A* (*1−Be^−kt^*)
Logistic	*Yt* = *A* (*1* + *Be^−kt^*)^−1^
von Bertalanffy	*Yt* = *A* (*1*−*Be^−kt^*)^3^
Richard’s	*Yt* = *A* (*1*−*Be^−kt^*)^m^

Where; *Yt* is the liveweight on t age, *A* is a mature liveweight, *B* is the proportion of mature weight which will reach after birth weight formed by Y0 and early t (the value of integral constants), *k* is the animal growth rate reach on mature liveweight, *e* denote natural exponential logarithm

## Results

Table-2 summarizes the key descriptive findings of the cattle population used in this study. To match the average daily gain (ADG) (Table-3) computed during those times, only the average birth weight and the 180 days (pre-weaning), 360 days (post-weaning), and 540 days (finishing) of age were included. Male BB crossbred offspring were more than 100% heavier (35.18 kg) than the KK male offspring (17.38 kg) for the average birthweight but not more than 2 kg heavier than BRAH’s (33.82 kg). Similar results were observed in the female group. During 180 days of age, BB crossbred male offspring remained more than 100% heavier (176.20 kg) than the KK (87.20 kg) but increased to almost 50% heavier than the BRAH (131.66 kg). For the female group, the BB crossbred offspring were 60% heavier (146.19 kg) than the KK (92.08 kg) and almost 20% heavier than the BRAH (122.0 kg). By 360 days of age, male BB crossbred offspring were almost 200% heavier (312.21 kg) than KK (106.16 kg) and almost 60% heavier than BRAH. The same phenomenon occurred in the female offspring, where the BB crossbred offspring were 100% heavier (246.20 kg) than those from KK (98.43 kg) but almost 50% heavier than BRAH (168.34 kg). At the finishing age of 540 days, for both male and female groups, BB crossbred offspring was more than 200% heavier (433.87 kg, male) (379.30 kg, female) than KK (136.59 kg, male) (127.66 kg, female) and 60%–70% heavier than BRAH (256.50 kg, male) (219.52 kg, female). According to Table-4, male and female BB crossbred offspring in the pre-, post-weaning, and finishing stages recorded an ADG value of more than 0.5 kg (male: 0.78 kg, 0.76 kg, and 0.68 kg, respectively) and (female: 0.63 kg, 0.55 kg, and 0.74 kg, respectively). However, for KK and BRAH, the highest ADG was only recorded during the pre-weaning stage and declined to 0.5 kg as they reached the finishing age. Table-3 shows the best-fit (highest R^2^, lowest MSE) non-linear regression growth model for Farm A (KK, male [R^2:^ 0.654, MSE: 448.667] and female [R^2:^ 0.691, MSE: 331.559]) and Farm B (BRAH male [R^2:^ 0.79, MSE: 1321.03] and female [R^2:^ 0.754, MSE: 874.538]) from the Brody model. On the other hand, the best-fit model for BB male and female crossbreds was the von Bertalanffy model (male R^2:^ 0.871, MSE: 2132.891) (female R^2:^ 0.806, MSE: 2233.176).

**Table-2:** Descriptive analysis of the offspring liveweight.

Age	Average						Standard deviation					
	
	Male			Female			Male			Female		
			
	BB	KK	BRAH	BB	KK	BRAH	BB	KK	BRAH	BB	KK	BRAH
0	35.18	17.58	33.82	32.64	16.35	30.07	5.68	1.17	6.42	5.91	1.10	4.62
180	176.20	87.20	131.66	146.19	92.08	122.0	50.32	16.52	37.43	40.81	19.57	20.01
360	312.21	106.16	195.38	246.20	98.43	168.34	68.14	25.61	32.42	62.00	19.60	33.32
540	433.87	136.59	256.50	379.30	127.66	219.52	54.36	27.33	74.25	32.53	19.88	29.55

**Age**	**Minimum**						**Maximum**					
	
	**Male**			**Female**			**Male**			**Female**		
			
	**BB**	**KK**	**BRAH**	**BB**	**KK**	**BRAH**	**BB**	**KK**	**BRAH**	**BB**	**KK**	**BRAH**

0	20.00	16.00	20.00	17.00	13.00	16.80	48.00	20.00	46.00	43.00	18.00	42.00
180	88.00	57.00	115.00	73.60	51.00	88.00	260.00	124.00	142.00	224.00	128.00	154.00
360	211.20	65.00	151.00	142.40	56.50	103.50	403.30	171.00	248.00	354.40	151.00	245.00
540	373.60	89.00	204.00	330.40	96.00	161.00	479.20	204.00	309.00	437.60	166.00	274.00

BB=Belgian Blue crossbreds, KK=Kedah-Kelantan, BRAH=Brahman

**Table-3:** The biological parameter estimates of the non-linear regression growth curves of the offspring.

Biological parameter	Brody	Brody	Brody	Brody	von Bertalanffy	von Bertalanffy
					
	KK (Male)	KK (Female)	BRAH (Male)	BRAH (Female)	BB (Male)	BB (Female)
R^2^ (Coefficient of determination)	0.654	0.691	0.79	0.754	0.871	0.806
MSE (Mean square error) residual	448.667	331.559	1321.03	874.538	2132.891	2233.176
A: Mature weight (kg)	142.48	119.69	428.05	251.89	527.49	518.49
B: Proportion of mature weight	0.837	0.843	0.916	0.873	0.592	0.575
k: Rate of maturing	0.04	0.06	0.001	0.003	0.003	0.003

KK=Kedah-Kelantan, BRAH=Brahman, BB=Belgian Blue

**Table-4:** The ADG of the offspring based on sex and breed.

ADG stage	ADG value					

	Male			Female		
	
	BB	KK	BRAH	BB	KK	BRAH
Pre-weaning	0.78	0.39	0.54	0.63	0.42	0.51
Post-weaning	0.76	0.10	0.35	0.55	0.03	0.26
Finishing	0.68	0.17	0.34	0.74	0.16	0.28

BB=Belgian Blue crossbreds, KK=Kedah-Kelantan, BRAH=Brahman

**Figure-3:**
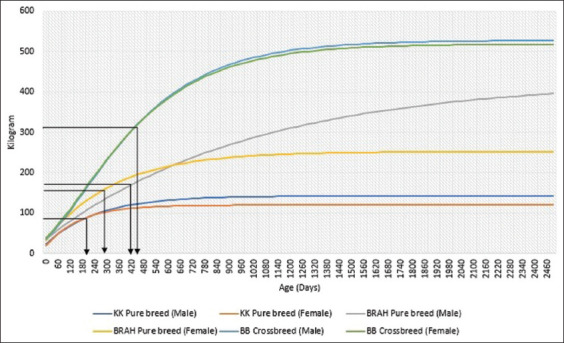
The non-linear regression growth curves of the offspring. KK=Kedah-Kelantan, BRAH=Brahman, BB=Belgian Blue. The black arrow ↴ indicates the inflection point based on liveweight (KG) and age (days).

For both models, emphasis was placed on the inflection points *A*, *B*, and *k*. The model growth curve’s inflection point corresponds to when cattle reach their fastest growth rate. The growth rate starts to slow down beyond this point as it ages. Based on Figure-3, *B. indicus* cattle, KK, and BRAH pure breeds reach an earlier inflection point than the *B. taurus* and *B. indicus* BB crossbreds (male and female offspring at 450 days, 300 kg liveweight). Later, growth rate gives a significant advantage to the BB crossbreds as it produces four times heavier *A* than the KK and two times heavier than the BRAH pure breed. The mature weight (B) proportion measures cattle growth compared with its maximum potential size or live weight. It is typically expressed as a percentage of the cattle’s mature weights. The cattle may be closer (higher percentage) to or further (lower percentage) from their mature weight, depending on their breed. From Table-3, the KK pure breed can be considered closer to its true potential due to its high B (male: 83.7% and female: 84.3%). The male BRAH pure breed attained the highest B (91.6%), suggesting its readiness to be bred or slaughtered. The female BRAH also possesses a high B (87.3%). In contrast, the BB crossbreds were considered far from ready for their maximum potential (male: 59.2% and female: 57.5%). This suggests that with correct farm management, the BB crossbreds would attain a heavier liveweight to generate more income. The *k* value is interpreted as the pace at which cattle grow and develop to reach their mature size or live weight. Cattle with a higher *k* may have a genetic advantage in reaching their mature size or live weight more quickly. However, they may also require more rigorous management and better nutrition to support their fast growth. The KK purebred has the highest *k* (male: 0.04, female: 0.06), in agreement with the inflection point mentioned before. On the other hand, BRAH purebreds (male: 0.001, female: 0.003) and BB crossbreds (male: 0.003, female: 0.003) have almost equivalent *k*, which explains their heavier liveweight at a later age.

The nonlinear regression growth curves of all offspring are presented in Figure-3. The nonlinear regression growth functions reflected by the growth curves are displayed in Table-5.

**Table-5:** The non-linear regression growth functions of Kedah-Kelantan pure breed, Brahman pure breed, and Belgian Blue crossbreds derived in this study.

Growth functions	Male	Female
Kedah-Kelantan pure breed (Brody)	Yt = 142.485 × (1−(0.837×e(−1×0.004×t)))	Yt = 119.696 ×(1−(0.843×e(−1×0.006×t)))
Brahman pure breed (Brody)	Yt = 428.05 × (1−(0.916×e(−1×0.001×t)))	Yt = 251.891 × (1−(0.873×e(−1×0.003×t)))
Belgian Blue crossbreds (von Bertalanffy)	Yt = 527.495 × (1−(0.592×e(−1×0.003×t)))^3^	Yt = 518.498 × (1−(0.575×e(−1×0.003×t)))^3^

e=mathematical exponential value, t=age

## Discussion

A strategy for enhancing the heterozygosity of the mh allele in beef cattle while minimizing homozygosity could result in leaner, more muscular carcasses [31]. Using tropical breeds for crossing with *B. taurus* breeds, known for their superior growth rates, generates enhanced finishing weights, according to previous reports [28] in the case of KK cattle. To crystalize experimental data on the performance of crossbred offspring generations, a suitable growth function should be employed to narrow down the data into significant biological parameters [22]. Smallholder beef cattle farmers rarely have the opportunity to comprehend the essential biological growth parameters due to a lack of knowledge, exposure, and educational background [32]. At the farm enterprise level, the decision-making process for live weight performance relies either on visual estimation [28] or the average market price [33]. Farmers will have limited power to dictate prices for their produce.

The crossbreeding of BB with *B. indicus* results in local beef cattle exhibiting the double-muscling trait and 50%–200% greater live weight from birth to 540 days. Livestock must gain more weight at every age stage to maintain a constant positive farm profit. Penasa *et al*. [34] revealed that 22 ± 7-day-old BB × Brown Swiss crossbred calves sold for €437, nearly 2.6 times the Brown Swiss pure breed’s €170. Thus, a heavier calf at such age can yield farmers a significant financial advantage.

The 234.98% ADG increase for KK males, 211.44% for females, 79.04% for male BRAHs, and 82.98% for female BRAHs in this study indicated double-muscling trait effects. The study indicated that KK had an ADG value below 0.5 kg/day for each growth phase (pre-weaning, post-weaning, and finishing). Farm B exhibited a superior ADG with BRAH pure-breed cattle. While Johari *et al*. [35] reported different findings, BRAH cattle are known to have superior growth compared to KK cattle. The study’s findings regarding the *B. indicus* breeds’ growth correspond with previous research [36, 37]. The BB crossbreeds, resulting from the crossbreeding of *B. indicus* and *B. taurus*, outperformed KK and BRAH pure breeds in this study. The higher ADG (234.98% over male KK, 211.44% over female KK, 79.04% over male BRAH, and 82.98% over female BRAH) from this study suggested double-muscling trait effects. Kadirveloo *et al*.[38] evaluated BB crossbred cattle’s performance and carcass traits. Crossbred cattle outperform purebred KK cattle in terms of live weights, dressing percentages, and carcass weights. Studies are needed to establish the impact of BB crossbreeding on KK cattle. Rollins *et al*. [10] revealed that crossbred calves with the double-muscled (m+) gene in the Charolais breed, in France, had an average daily weight gain of 1.14 kg, which was 2% higher than those without the gene. A study by Bittante *et al*. [8] tested the crossbreeding of Brown Swiss dairy cows with BB or Piedmontese sires, finding larger ADGs in the former (1.22 kg) compared to the latter (1.14 kg), with this difference proven significant at p < 0.05. Calves sired by BB bulls and born to dams of Brown Swiss, Simmental, and Rendena breeds were assessed. At 90 days of age, the ADG and liveweight for calves born from the dams of BS, Si, and Ren were 1.47 kg (274 kg), 1.45 kg (273 kg), and 1.49 kg (274 kg) [39]. The study found that BB-sired calves, irrespective of their dam breed, displayed an ADG value above 1.0 kg, a level associated with high and consistent performance in BB purebred farming, according to Fiems [6]. A group of 90-day-old BB and BRAH first-generation crossbred calves raised in Indonesia was reported to have a mean ADG of 0.70 ± 0.15 kg and liveweight of 95.10 ± 12.07 kg, which is heavier than the same-rearing group of 90-day-old Wagyu and BRAH first-generation crossbred calves with a mean ADG of 0.64 ± 0.17 kg and liveweight of 90.91 ± 27.70 kg [40]. The BB crossbreds recorded ADGs of over 0.7 kg for males and 0.64 kg for females in this study, indicating comparable adaptability to tropical climates in Indonesia and Malaysia. In this study, a BB crossbred male calf’s liveweight at 180 days of age was recorded to be 176.20 kg, surpassing the weights of BB and Holstein crossbred male calves reported by Akbas *et al*. [41]. Genetic distance between breeds, as in *B. taurus* × *B. indicus* versus *B. taurus* × *B. taurus*, significantly enhances growth performance through heterosis [42].

This study employed Brody, Logistic, von Bertalanffy, and Richard’s functions among the most commonly used non-linear regression growth functions for their practicality and applicability in explaining beef cattle growth concepts [22]. The Brody model provided the best fit for the non-linear regression growth functions of the *B. indicus* KK (142.48 kg male, 119.69 kg female) and BRAH (428.05 kg male, 251.89 kg female) breeds, while the von Bertalanffy model had the best fit for the BB crossbreds (527.49 kg male, 518.49 kg female). According to Teleken *et al*. [22], the Brody model was the best fit for the Nellore cow, while the von Bertalanffy model was best for the Holstein-Friesian bull. The best-fit Brody growth model, determined by Amrullah *et al*. [21] through non-linear regression analysis (R^2^: 0.90, A: 507.19 ± 8.27 kg), explains BRAH cows. The estimated mature liveweight of the KK breed in this study by its best-fit model (Brody) is considered the lowest among the other breed groups because of its natural fast maturation [43]. The BB breed attains growth maturity later in life [44]. Among all breed groups, this one has the greatest mature liveweight. The temperate breed’s longer time to maturity results in a heavier liveweight, beneficial to farmers and decision-makers. Zimmermann *et al*. [45] compared growth rates of cows sired by BB, Angus, and Tuli breeds and found no statistical difference in maturing rate (k) between BB and Angus (p = 0.13), but a faster k for BB than Tuli (p = 0.02). The study found that *B. taurus* breeds reached puberty at a slightly younger age (0.56–0.58) than *B. indicus* breeds (0.60). The study by Zimmermann *et al*. [45] determined mature weights of 313.80 kg (at weaning, 180 days) and 609.0 kg (at maturity, 6 years) for BRAH-influenced cows using a non-linear regression quadratic function. The 180-day BRAH female from Farm B yielded 122.0 kg, less than that from this study. Differences in breed adaptability may account for variations in this phenomenon across diverse climates. The breed’s effect on maturity significantly influences numerous growth traits, often resulting in discernible differences [25]. The nonlinear regression growth functions in Table-5 serve as a useful tool for farmers and researchers to estimate the liveweight and age of beef cattle from similar breed groups.

The genetic and expressional factors of BB crossbreds determine the degree of double muscling [46]. In Malaysia, farmers weigh the pros and cons of implementing double-muscled animals in various farming techniques. Muscular hypertrophy in double-muscled animals can provide insights into the relationship between cow breeding features and slaughter traits. Crossbreeding beef cattle with double-muscling traits can enhance meat yield and self-sufficiency [6]. Making beef supply more predictable and plentiful would stabilize prices [47], reducing food import bills and dependency on imports [33]. Enhancing beef production efficiency would secure a consistent income source for farmers and keep them in the industry for a longer period. Introducing double-muscling genes into tropical beef herds on these grounds would significantly bolster long-term food security and sustainability for the beef farming business. Beef cattle smallholder farming in Malaysia ensures food security and contributes as a group of small and medium enterprises, promoting macroeconomic progress at a national level. Assessing beef cattle growth using the trait of double-muscling leads to increased beef production.

From fieldwork data, this beef cattle growth study yields a significant understanding of the determinants impacting cattle growth and efficiency. This study’s limitations should be taken into account when analyzing its findings. The sample size was limited by practical considerations. Due to limited resources and time, only a certain number of cattle were observed and measured despite attempts to include all possible cattle. The accuracy of the conclusions depends heavily on the quality of the field data. Meticulous data collection efforts may still result in incomplete or inaccurate measurements. Real-world data collection can present difficulties and yield errors. Analyzing field data involves dealing with the intricacies of multiple interacting variables. The study applied robust statistical methods to minimize the impact of hidden factors on the results and strengthen the validity of the findings.

## Conclusion

The mature weights of various beef breeds were estimated under comparable conditions using Brody, Logistic, von Bertalanffy, and Richard’s regression growth models. This study’s non-linear regression growth functions revealed that KK and BRAH purebreds grew differently than BB crossbreds, most notably in their pre-weaning to peak weight growth rates. An effective explanation of these traits cannot be achieved through descriptive analysis. The BB crossbred offspring outperformed the KK and BRAH offspring in every liveweight measurement, including birth weight, 180 days weight, 360 days weight, 540 days weight, pre-weaning ADG, post-weaning ADG, finishing ADG, and mature weight estimation. The BB crossbred offspring weighed over 50% more than the BRAH pure breed and over 200% more than the KK pure breed on each indicator. The Brody model was suitable for explaining the growth of KK and BRAH pure breeds, while the von Bertalanffy model was more appropriate for BB crossbreds. To ensure BB crossbreeding is economically viable, significant farm operations aspects must be taken into account.

## Authors’ Contributions

UNA and GYM: Conceptualized and designed the study. UNA and GYM: Prepared the materials and data collection and analysis. UNA: Drafted the manuscript. Both authors have read, reviewed, revised, and approved the final manuscript.
